# Approaches to liquid chromatography tandem mass spectrometry assessment of glyphosate residues in wine

**DOI:** 10.1007/s00216-021-03775-w

**Published:** 2021-11-25

**Authors:** L. Pérez-Mayán, G. Castro, M. Ramil, R. Cela, I. Rodríguez

**Affiliations:** Department of Analytical Chemistry, Nutrition and Food Sciences. Research Institute On Chemical and Biological Analysis (IAQBUS), Universidade de Santiago de Compostela, Santiago de Compostela, 15782 Spain

**Keywords:** Glyphosate, Wine, Liquid chromatography tandem mass spectrometry, Derivatization, Direct injection

## Abstract

The performance of two different analytical methodologies to investigate the presence of glyphosate (GLY) and aminomethylphosphonic acid (AMPA) residues in wine samples was evaluated. Transformation of compounds in their fluorene-9-methyloxycarbonyl derivatives permitted their separation under reversed-phase liquid chromatography with tandem mass spectrometry (LC–MS/MS) determination. Although the wine matrix severely impaired the efficiency of GLY derivatization, this drawback was solved using a molecularly imprinted sorbent for the previous, selective extraction of GLY and AMPA from wine. Alternatively, the use of a strong anionic exchange, polyvinyl alcohol-based LC column, turned to be the most effective alternative for direct determination of both compounds in diluted wine samples. The chromatographic behavior of this column and the magnitude of matrix effects observed during analysis of diluted wine samples were significantly affected by the composition of the mobile phase. Under final working conditions, this column permitted the separation of AMPA and the fungicide fosetyl (which shows common transitions in tandem MS/MS methods), it improved significantly the sample throughput versus extraction-derivatization-purification method, and it allowed the use of solvent-based calibration standards. Both analytical procedures provided similar limits of quantification (LOQs) for GLY (0.5–1.0 ng mL^−1^), while the multistep method was 8 times more sensitive to AMPA than the direct procedure. GLY residues stayed above method LOQs in 70% of the processed wines; however, concentrations measured in 95% of positive samples remained 100 times below the maximum residue limit (MRL) set for GLY in vinification grapes.

**Graphical abstract:**

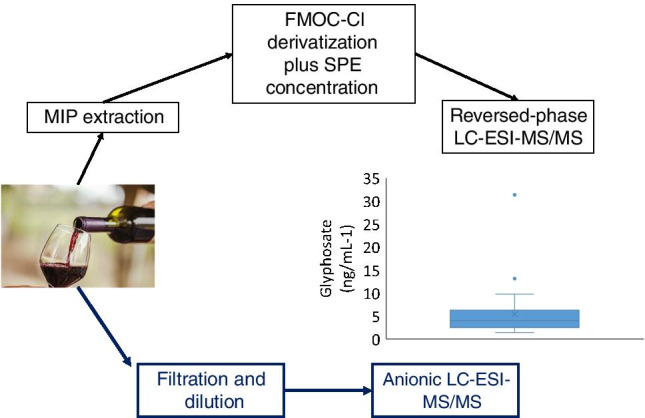

**Supplementary Information:**

The online version contains supplementary material available at 10.1007/s00216-021-03775-w.

## Introduction

Glyphosate (GLY) is worldwide employed as a non-selective weed killer in gardening and agriculture [1]. With the same aim, the limits of roads are often fumigated with this herbicide. The development of genetically modified (GMO) plants was a milestone in the use of GLY, allowing a significant reduction in the production costs of cotton, soya, and maize, among others. On the other hand, this application is known to lead to GLY residues in the obtained seeds and fibers [2–4]. Other uses which might contribute to introducing GLY in the food chain are the control of herbaceous vegetation in permanent crops (i.e., vineyards) and, particularly, the application of GLY as plant desiccant, to homogenize the harvest of crops. Despite that GLY applications are still authorized in most countries [5], there is an increasing concern in relation to the medium-term environmental and toxicological effects of the parent herbicide and aminomethylphosphonic acid (AMPA), the main metabolite of GLY, in the environment.

The quantification of trace concentrations of GLY and AMPA represents a challenging issue due to the high polar, zwitterion character, low molecular weight, and ligand properties of both species. Derivatization of polar moieties in the structure of both compounds (i.e., using a combination of trifluoroacetic acid and trifluoroethanol [6], or a silylation reagent [7]) allows their determination by gas chromatography-mass spectrometry (GC–MS) [6, 8]. However, liquid chromatography-mass spectrometry (LC–MS) remains the most resorted technique for the quantification of GLY and AMPA in environmental and food samples. In this case, derivatization of the amino moiety with 9-fluorenylmethyl chloroformate (FMOC-Cl), which can be performed in aqueous media, makes possible the retention and separation of both compounds in reversed-phase LC columns [9].

In order to simplify and to fast the monitoring of GLY and AMPA, researchers have addressed the development of more efficient and/or selective extraction procedures, such as molecularly imprinted polymers (MIP), so far tested for extraction and concentration of GLY from water samples [10]; strategies to mask the ligand character of GLY and AMPA [11]; and particularly, novel stationary phases (based on hydrophilic interactions, HILIC [12, 13], ionic exchange [14, 15], or mixed-mode columns [16]) in LC improving the retention of these compounds and permitting the use of mobile phases compatible with electrospray ionization, previously to their mass spectrometry detection (ESI–MS).

The determination of GLY and AMPA in alcoholic, fermentation drinks, as it is the case of wine, has some additional particularities. First, in-sample derivatization using FMOC-Cl is more complex than in the case of environmental aqueous matrices given the relevant content of amino acids in this beverage and sample component precipitation (particularly in case of red wines) when wine is adjusted at pH values required to complete the reaction (ca., 9 units). Second, the potential residues of GLY and/or AMPA are expected to stay at lower levels than in other beverages and foodstuffs[17], since (1) application of the herbicide to vineyard soils is mainly limited to the end of winter, during the non-vegetative period of vines; and (2) the extremely low tolerance of vines to herbicidal preparations containing GLY. Another issue to consider during wine analysis is the possible interference of fosetyl (the ethylphosphonate derivative of the fungicide fosetyl-aluminum) with AMPA using LC–ESI–MS/MS methods, unless both compounds are properly separated, during either sample preparation or LC determination.

Herein, we explore the analytical features of two different approaches to determine GLY and AMPA residues in red and white wine samples by LC–MS, based on the use of triple quadrupole instruments. The first alternative involves a fine tuning of the FMOC-Cl derivatization method after compounds extraction from wine samples using a MIP sorbent. The second approach considers direct analysis of native compounds employing mixed-mode (hydrophilic interaction, HILIC, and weak anion exchange column) and strong anionic exchange columns; moreover, it includes the simultaneous determination of Fosetyl (FOS). This compound is not amenable to FMOC derivatization and its presence in wines might be due to transformation of the parent fungicide (Fosetyl-Aluminum), as well as to the reaction between ethanol and phosphorus acid derivatives employed as fertilizers [18]. Developed methods were applied to investigate the presence of targeted compounds in commercial wines in order to understand the potential contamination with GLY and/or AMPA and the transfer of FOS residues from grapes to wine.

## Material and methods

### Standards and solvents

Standards of GLY and AMPA were purchased from Sigma-Aldrich (St. Louis, MO, USA). Fosetyl-aluminum and GLY-FMOC were purchased from Dr. Ehrenstorfer (Augsburg, Germany). Isotope-labelled GLY (1,2-^13^C,^15^ N), AMPA (^13^C^15^N), and fosetyl-aluminum-d_15_ were acquired from Toronto Research Chemicals (North York, Canada). Reagent-grade disodium tetraborate decahydrate (> 99.5%) was obtained from Merck (Darmstadt, Germany). FMOC-Cl, for HPLC derivatization, and citric acid (> 99.5%) were purchased from Sigma-Aldrich (St. Louis, MO, USA). Formic acid reagent grade (> 98%) and hydrochloric acid solution 0.1 M were provided by Scharlab (Sentmenat, Spain). Ammonium bicarbonate eluent additive for LC–MS (> 99.5%) was acquired from Honeywell Fluka (Seelz, Germany). Methanol (MeOH) LC–MS purity was provided by Fisher (Geel, Belgium). Acetonitrile (ACN) LC–MS purity, sodium hydroxide, and dichloromethane (DCM) were supplied by Merck (Darmstadt, Germany). Ultra-pure deionized water (18.2 MΩcm^−1^) was obtained from a Genie U system (Rephile, Shanghai, China).

Molecular-imprinted (250 mg/3 mL AFFINIMIP® SPE Glyphosate-AMPA) and reversed-phase OASIS HLB 200-mg cartridges were acquired from AFFINISEP (Petit-Couronne, France) and Waters (Milford, MA, USA), respectively.

### Samples and sample preparation

#### Determination of GLY and AMPA as FMOC derivatives

Unfiltered wine samples (2 mL) were spiked with labelled analogues of GLY and AMPA (10 ng mL^−1^). Thereafter, they were adjusted at pH 7 and diluted to 10 mL with ultrapure water. The first step in the sample preparation scheme consisted of GLY and AMPA extraction from the wine matrix using the MIP sorbent. Cartridges were first conditioned with ultrapure water. After loading the diluted beverage, the sorbent was rinsed with ultrapure water and analytes were eluted with 10 mL of hydrochloric acid 0.1 M. This extract was neutralized, using NaOH 0.1 M aqueous solution, previously to perform the derivatization reaction with 1 mL of borate 40 mM buffer and the same volume of a FMOC-Cl 6.5 mM solution in ACN. The reaction was stopped after 2 h using 0.5 mL of formic acid. The final step was a solid-phase extraction clean-up and concentration of FMOC-derivatized compounds using an Oasis HLB 200 mg cartridge. Conditioning and washing solvents were MeOH and ultrapure water, respectively. The sample  was passed through the cartridge, and it was washed with ultrapure water and DCM (to remove excess of FMOC-Cl and its hydrolysis derivatives) before elution using 2 mL MeOH. The reversed-phase cartridge extract was concentrated to dryness and reconstituted with 200 µL of MeOH:ultrapure water (1:1) 0.5% formic acid, filtered (0.22 µm) and injected in the LC–MS/MS system.

#### Direct determination of GLY, AMPA, and fosetyl

Wine (1 mL) was spiked with isotopically labelled analogues of GLY, AMPA, and FOS (10 ng mL^−1^), passed through a 0.22-µm syringe filter, and diluted ten-fold with ultrapure water before LC–ESI–MS/MS analysis.

### Equipment and determination conditions

FMOC-derivatized compounds were determined using a LC–MS/MS XEVO TQD, triple quadrupole mass spectrometer, acquired from Waters (St. Louis, MO, USA) and furnished with a Z spray ESI source, working in positive mode. GLY and AMPA were separated with a Waters (Milford, MA, USA) Acquity BEH C18 (50 mm × 2.1 mm, 1.7 µm) reversed-phase column connected to a C18 2.1 mm i.d. guard cartridge from Phenomenex (Torrance, CA, USA). Further details of mobile phase composition and gradient are provided as supplementary material, Table S1. The injection volume was 5 μL. Nitrogen was employed as drying gas in the ESI source (450 °C at 1000 L h^−1^). The ESI needle was maintained at 1.5 kV.

A second LC–MS/MS, consisting of an Agilent 1290 Infinity II connected to a triple quadrupole (QqQ) mass analyzer (MS), Agilent 6495, through a jet stream ESI source, was used for the determination of free compounds. Two different columns were investigated for compound separation: a mixed-mode (HILIC and weak anionic exchange) Torus DEA column (100 mm × 2.1 mm, 1.7 µm), and a strong anionic exchange-type Metrosep A Supp column (150 mm × 2.1 mm, 5 µm), on-line connected to a guard cartridge with the same stationary phase. Table S1 summarizes the different tested mobile phases in combination with each of these two columns. In both cases, the injection volume (1:10 diluted wines or ethanolic aqueous solutions, 12:88, v:v) was 25 µL. Nitrogen was employed as nebulizing (55 psi), drying (150 °C, 11 L min^−1^) and sheath gas (400 °C, 12 L min^−1^) in the ESI source. Voltages of the ESI source were 3000 and 1500 V for positive and negative ionizations, respectively.

The trend of polar compounds such as GLY and AMPA to bond metal ions present in the instrument connections and columns is widely recognized. Thus, whatever the LC–MS/MS model and determination approach (as FMOC derivatives or as free compounds), a daily cleaning of the employed LC system was performed to ensure the chelation of possible interferences [19]. For this purpose, 5 mM citric acid aqueous solution flows through the LC modules at 0.5 mL min^−1^ for 30 min, from the binary pump to the ESI source, with a death volume situated in the column position.

The procedural limits of quantification (LOQs) of the analytical approaches described in this study, established for S/N of 10, were estimated from responses obtained for the lowest level matrix-matched calibration standard (case of FMOC-derivatized species) or from solvent-based standards (diluted in ethanol:water, 12:88), corrected with matrix effects observed for red and white wines. In addition, according to the SANTE/12682/2019 guidelines [20], at compounds LOQs the ratio between quantification and qualification transitions must match the average value observed for the rest of the levels in the calibration curve, within a ± 30% maximum variation.

## Results and discussion

### Optimization and characterization of GLY and AMPA determination as FMOC derivatives

**Fig. 1:**
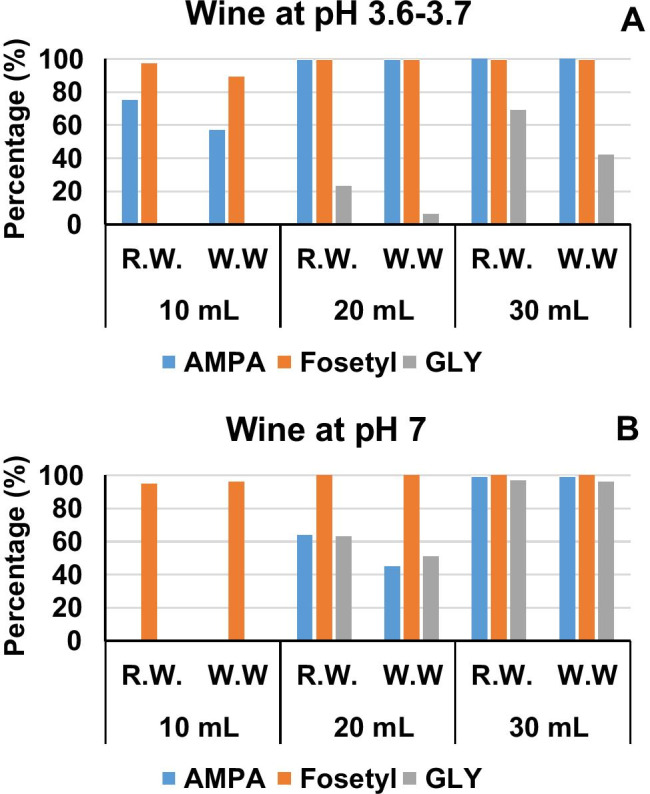
Average breakthrough percentages (*n* = 2 replicates) for AMPA, GLY, and fosetyl in the MIP sorbent corresponding to 1:5 diluted red (R.W.) and white wine (W.W.) as function of loaded sample volume and pH. **A** Data at typical wine pH (3.6–3.7). **B** Data for samples adjusted at pH 7

Conditions employed for derivatization and extraction of GLY and AMPA, as FMOC derivatives, from water samples have been described in previous studies [9, 21]. Herein, we found that those conditions were not compatible with the wine matrix, particularly in the case of red wines. At the pH value required to carry out the reaction (ca., 9 units), many compounds present in the wine matrix tend to precipitate (Fig. S1A and S1B), which might compromise the efficiency of the derivatization process. To solve this shortcoming, we investigate the suitability of MIP SPE cartridges to isolate both compounds from wine samples. To this aim, samples (red and white wines) were diluted 1:5 with ultrapure water, spiked with target compounds, at high level (500 ng mL^−1^), and passed through MIP sorbents. Figure 1 shows the percentage of breakthrough for GLY, AMPA, and fosetyl as a function of the sample volume and pH. The MIP sorbent showed a high affinity for GLY considering a loading volume of 10 mL; however, AMPA could be retained only after increasing the pH of the diluted wine solution to 7 units. Higher pH values were not tested to prevent precipitation of the wine matrix. At the investigated pHs, fosetyl was not retained by the MIP sorbent. This fact prevents the use of the MIP sorbent for the simultaneous extraction of the three compounds. On the other hand, it might be useful to avoid the isobaric interferences of fosetyl in the LC–MS/MS determination of AMPA (as free compounds), if they are not baseline separated by the LC column. As regards the elution step, compounds were recovered from the polymer with 10 mL of an aqueous solution of hydrochloric acid (0.1 M) [10]. As shown in the supplementary information (Fig. S1A to S1D), the MIP extraction step reduced the visual complexity of the wine matrix compared to the raw sample.

Derivatization conditions were assessed considering different concentrations of FMOC-Cl prepared in ACN and different reaction times. A 1:10 ratio between the FMOC-Cl solution in acetonitrile and the MIP extract was maintained in all the assays [9]. Firstly, increased FMOC-Cl concentrations (1, 6.5, and 12 mM) were tested to achieve the best derivatization efficiency. Results show that neither a defect nor excess of FMOC-Cl was adverse; thus, the intermediate level was selected. Regarding the derivatization time, tests were carried out in the time range from 0.5 to 4 h. It was noticed that no significant signal improvement was reached for reaction times over 2 h (Fig. S2).

After stopping the FMOC derivatization reaction, removing the excess of FMOC-Cl and its hydrolysis by-products is recommended. To this end, the HLB cartridge was rinsed with DCM [6]. The employed volume was 2 mL. FMOC derivatives were recovered with 2 mL of MeOH, and no losses were noticed during dryness concentration of this extract.

In order to better understand the effect of the MIP pre-extraction step in the performance of compound derivatization, we compared the responses (peak areas without internal surrogate correction) for aliquots of a white wine spiked at the same concentration (20 ng mL^−1^) submitted to above derivatization-concentration conditions, with and without MIP pretreatment. Responses obtained for AMPA in MIP extracted wine samples were nearly twice those observed following the conventional (direct) FMOC derivatization approach. In case of GLY, 20-times higher peak areas were noticed when combining MIP extraction with FMOC derivatization. Fig. S3 shows the normalized peaks areas for the FMOC derivatives of both compounds without MIP pre-extraction, versus those observed following this procedure. In a further experiment, responses obtained for a commercially available standard of GLY-FMOC added to final extracts from an ecological wine, which were obtained using the above described approaches, were compared to those observed for a reference standard in MeOH:H_2_O (1:1). Signal suppression (calculated as 1 minus the ratio between peak areas for matrix-matched and solvent based GLY-FMOC standards) accounted for 30% and 15% for in-sample derivatization and MIP extraction followed by FMOC derivatization, respectively. Thus, the poor performance of the direct (in sample) FMOC derivatization approach for GLY (Fig. S3) was mostly related to an impairment of the reaction due to the wine matrix, rather than to matrix effects in the ESI source.

### Performance of FMOC derivatization method

The LC–MS determination of derivatized AMPA and FMOC can be addressed either using positive or negative ESI modes. Under conditions reported in section “Equipment and determination conditions” and Table S1, we found that the first mode presented lower LOQs than the second one. The quantifying (Q1), qualifying (Q2), and other *m/z* corresponding to product ions of AMPA and GLY as FMOC-derivatives are summarized in Table 1. Despite that the transition from [M + H]^+^ precursors to product ion at *m/z* 179.0 (corresponding to the fluorenyl methyl moiety in the derivatization reagent) was the most intense, it showed lower signal to noise (S/N) ratios than others leading to most specific product ions associated to the molecules of AMPA and GLY. For this reason, it is not advisable to consider transitions to product ions at *m/z* 179.0, neither for quantification nor as primary qualifying transition. Fig. S4 shows the structures assigned to most intense product ions in the ESI ( +) spectra of AMPA and GLY as FMOC derivatives; see Table 1.

**Table 1:** LC-ESI ( +)-MS/MS conditions for AMPA and GLY determination as FMOC derivatives

Compound	Retention time (min)	Precursor ion	Cone voltage (V)	Q1 (CE)	Q2 (CE)	Q2/Q1 ratio	Other ions (CE)
AMPA	6.04	334	20	156.0 (10)	112.0 (15)	0.98	179.0 (20)
GLY	5.69	392	20	88.0 (20)	214.0 (10)	0.35	170 .0 (15); 179.0 (25)
AMPA-^13^C^15^N	5.96	336	20	158 (10)	114.0 (15)	0.95	179.0 (20)
GLY-^13^C_2_^15^N	5.61	395	20	91.0 (20)	217.0 (10)	0.53	173.0 (15); 179.0 (20)

Linearity was investigated using matrix-matched standards obtained from aliquots of ecologic production wines spiked with increasing concentrations of AMPA and GLY in the range from 1 to 100 ng mL^−1^ (*n* = 7 levels) and a constant level of AMPA-^13^C^15^N and GLY-^13^C_2_^15^N (10 ng mL^−1^). Responses (peak areas for MRM transitions) corrected with those measured for SSs were plotted versus added concentrations. Determination coefficients (*R*^2^) of the obtained graphs stayed above 0.998 for both compounds (Table 2). Accuracy of the method was investigated with four samples, each processed in triplicate, spiked at two different concentration levels: 10 and 25 ng mL^−1^. Global recoveries varied in the range from 99 to 117%, and from 91 to 107% for GLY and AMPA, respectively (Table 2).

**Table 2:** Performance of LC–ESI–MS/MS for GLY and AMPA determination after MIP extraction and FMOC derivatization

Compound	Linearity (*R*^2^, 1–100 ng mL^−1^, *n* = 7 levels)	Recovery (standard deviation, *n* = 3 replicates)				LOQ ng mL^−1^
		Red wine		White wine		
		10 ng mL^−1^	25 ng mL^−1^	10 ng mL^−1^	25 ng mL^−1^	
AMPA	0.9985	103 (20)	106 (2)	91 (10)	107 (9)	1
GLY	0.9992	99 (5)	107 (6)	117 (9)	106 (6)	0.5

### Direct determination of GLY, AMPA, and fosetyl

**Fig. 2:**
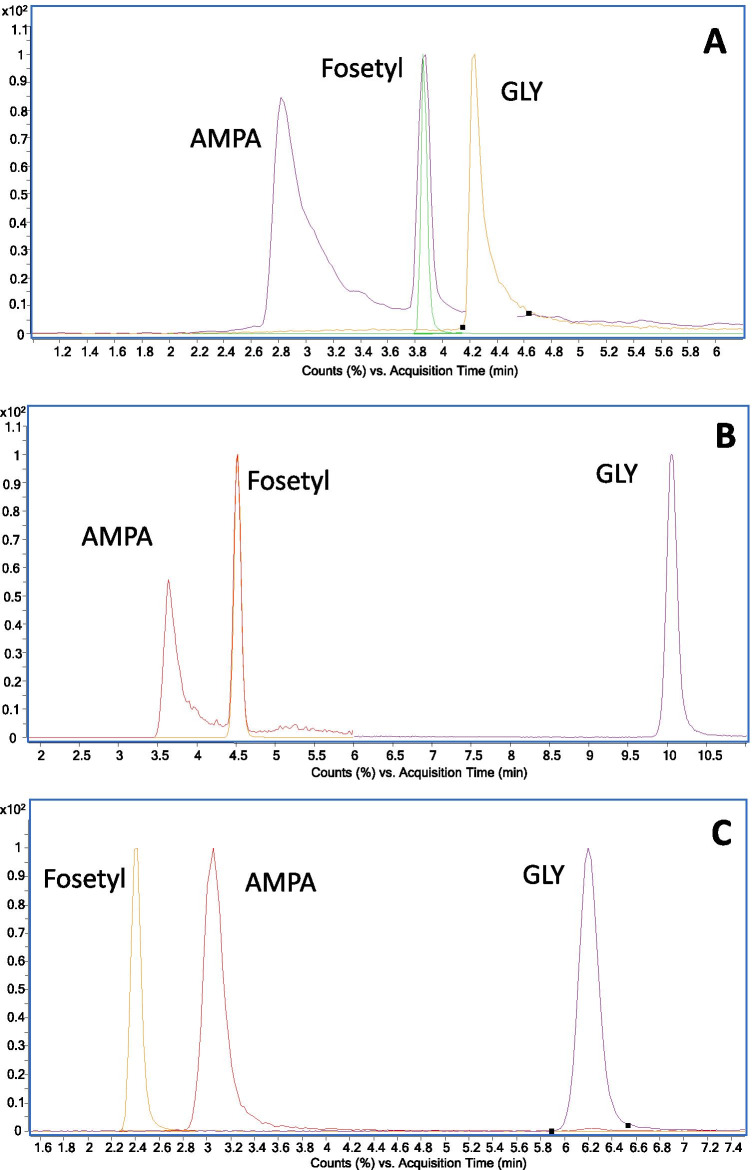
LC–ESI–MS/MS (MRM) chromatograms for the quantification transitions of AMPA, fosetyl, and GLY corresponding to a standard in ethanol:water (12:88). **A** Mixed-mode column. **B** Anionic exchange column, gradient 1. **C** Anionic exchange column, gradient 2. Concentration level: 250 ng mL^−1^ (**A**), 25 ng mL^−1^ (**B** and **C**)

**Fig. 3:**
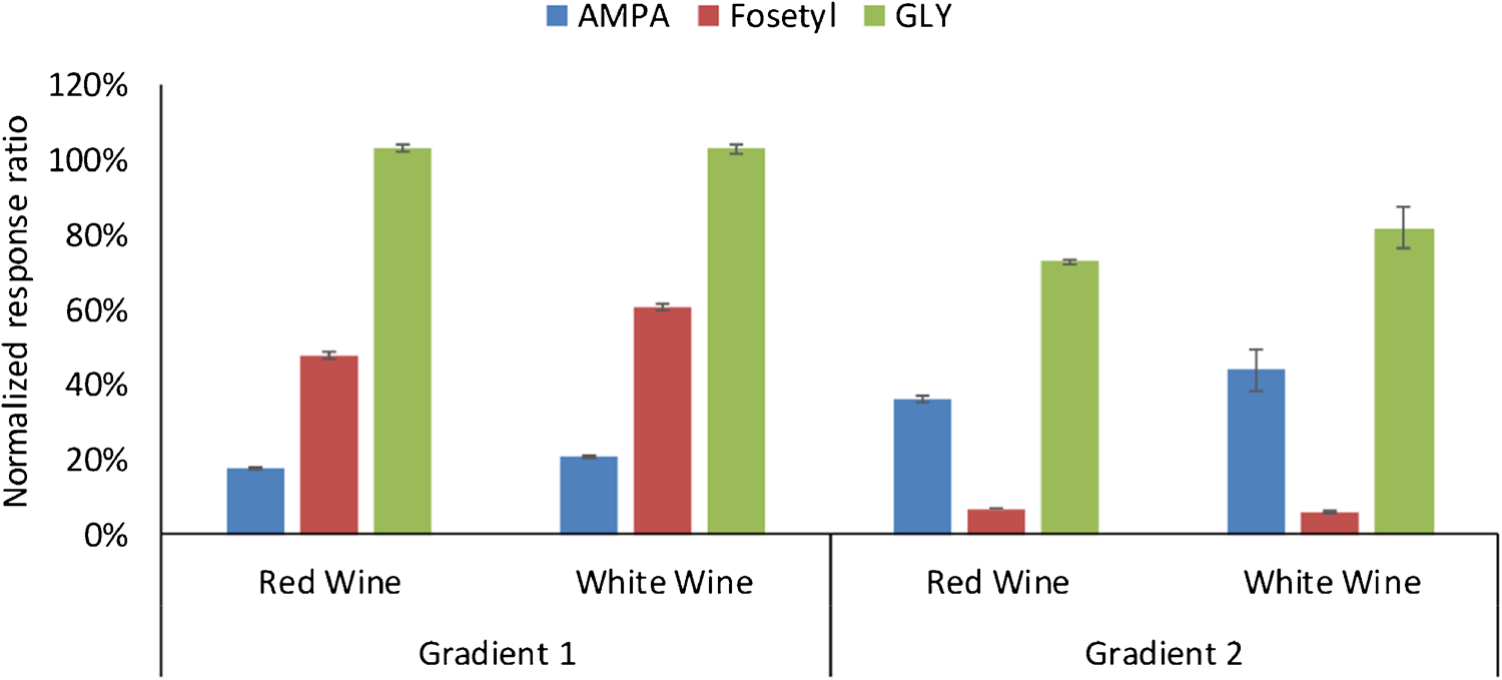
Normalized response ratios of slopes corresponding to calibration curves (0.5–100 ng mL^−1^, *n* = 6 levels) for wine samples versus ethanol:water (12:88) solutions

Success of compounds determination as free species depends mainly on the efficiency of their LC separation (peak shape and separation between AMPA and Fosetyl), and the effect of the wine matrix in the efficiency of their ionization. Figure 2 shows the chromatograms obtained for target compounds using the mixed-mode (Fig. 2A) and the strong anionic exchange column (Fig. 2B and C) considering mobile phase compositions and gradients compiled in Table S1. Selected columns and mobile phases have been previously reported for AMPA and GLY determination in water samples [15, 22]. In the three cases, separations were carried out in the same LC–ESI–MS/MS instrument, under identical conditions as regards ESI parameters and MRM transitions (Table 3). A symmetric, narrow peak was obtained for fosetyl under every investigated condition (Fig. 2). On the other hand, the mixed-mode column provided non-symmetric and wider peaks for AMPA and GLY (Fig. 2A) than the polyvinyl alcohol strong anionic exchange one (Fig. 2B and C). Some attempts to improve peak shape were made including medronic acid (5 µM) in the aqueous phase used in combination with the mixed-mode column [11]; however, no changes were observed in the chromatographic profiles of both herbicides. For the same concentration solvent-based standard, much lower S/N ratios were noticed for the peaks of AMPA and GLY obtained using the mixed-mode column than employing the polyvinyl alcohol one. Thus, the latter was considered for further experiments. As observed (Fig. 2B and C), the elution order between AMPA and fosetyl varied depending on the employed mobile phase. The retention of the compounds was slightly higher using gradient 1 (Table S1) (Table 3). Fig. 3 shows the normalized responses corresponding to the ratio between slopes of calibration curves for spiked wine samples (diluted 1:10 with ultrapure water) and ethanol:water (12:88) solutions, in the range from 0.5 to 100 ng mL^−1^ (referred to non-diluted matrix, *n* = 6 levels) as a function of the employed gradient. Graphs were built plotting the peak area for the quantification ions, without internal surrogate correction, versus concentration. Values below 100% point out to suppression in the efficiency of the ESI ionization for wine samples versus solvent-based standards, while a normalized ratio of 100% means absence of signal suppression. Normalized response ratios for AMPA and fosetyl varied greatly depending on the employed gradient. As a general statement, the shorter their retention times, the most significant the signal suppression effects. Thus, the lower normalized response ratios for AMPA corresponded to gradient 1 and those for fosetyl to gradient 2. In case of GLY, moderate suppression of its ionization efficiency was only noticed with gradient 2.

**Table 3:** LC-ESI ( ±)-MS/MS conditions for AMPA, GLY, and fosetyl determination as free compounds using the strong anionic exchange column

Compound	Retention time (min)		ESI mode	Precursor ion	Q1 (CE)	Q2 (CE)	Q2/Q1 ratio	Other ions (CE)
	Gradient 1	Gradient 2						
AMPA	3.60	3.03	-	110	63 (20)	79 (36)	0.94	
GLY	9.92	6.17	+	170	88 (8)	60 (18)	0.28	42 (32)
Fosetyl	4.54	2.39	-	109	81 (12)	63 (36)	0.35	79 (28)
AMPA-^13^C^15^N	3.61	3.02	-	112	79 (36)	63 (20)	1.05	
GLY-^13^C_2_^15^N	9.92	6.17	+	173	91 (8)	62 (17)	0.30	
FOS- d_15_	4.52	2.38	-	114	63 (36)	81 (12)	0.002	79 (28); 83 (12)

Table 4 summarizes the determination coefficients (*R*^2^, 0.5–100 ng mL^−1^) for solvent-based standards after IS correction, the instrumental LOQs of the system, and the procedural LOQs (estimated for red wine), calculated considering signal suppression effects observed for each compound as function of the employed gradient. The injected volume was 25 µL. Whatever the considered gradient, *R*^2^ values above 0.996 were obtained within the tested calibration range. The instrumental LOQs, estimated for solvent-based standards, were similar for both gradients, except in the case of AMPA. For this compound, a twice lower LOQ was obtained using gradient 2 (Table 4). In case of procedural LOQs, obtained values referred to red wine, ranged from 0.4 to 8.3 ng mL^−1^, depending on the compound and the employed gradient (Table 4). Lower procedural LOQs for fosetyl and GLY were noticed using gradient 1 (which was selected to continue the study), whereas for AMPA gradient 2 performed better in terms of achieved LOQ. For this compound, the procedural LOQs attained with the direct determination method were higher than that corresponding to FMOC derivatization, while similar LOQs were found for GLY using any of both strategies (Table 2 and Table 4).

**Table 4:** Linearity, instrumental, and procedural LOQs (ng mL^−1^), and recovery values obtained for AMPA, fosetyl, and GLY using the strong anionic exchange column with gradient 1

Compound	*R*^2^ (0.5–100 ng mL^−1^, *n* = 6 levels)		Instrumental LOQs		Procedural LOQs		Recoveries (%, with SD, *n* = 3 replicates)^a^			
	Gradient 1	Gradient 2	Gradient 1	Gradient 2	Gradient 1	Gradient 2	R.W		W.W	
							10 ng mL^−1^	25 ng mL^−1^	10 ng mL^−1^	25 ng mL^−1^
AMPA	0.9961	0.9998	1.5	0.8	8.3	2.8	91 (3)	113 (8)	115 (9)	122 (6)
Fosetyl	0.9998	0.9998	0.2	0.2	0.4	3.3	90 (1)	98 (7)	90 (2)	106 (8)
GLY	0.9985	0.9994	1	1	1.0	1.4	94 (3)	103 (3)	111 (9)	106 (8)

^a^ Recoveries obtained using gradient 1

The accuracy of direct determination was evaluated with spiked fractions of two different wines, using solvent-based standards containing same concentration of ISs. For each wine matrix, non-spiked aliquots and fractions fortified at concentration levels of 10 and 25 ng mL^−1^ were injected in triplicate. Recoveries were defined as the difference of concentrations measured in spiked and non-spiked fractions of each sample divided by the added value. The obtained values varied between 90 and 122%, with standard deviations below 10% (Table 4).

### Assessment of residues in wine samples

A set of 44 commercial wines from 2018 to 2020 campaigns (10 white and 34 red wines), acquired from local supermarkets, were processed in order to investigate the occurrence of target species. A group of 17 samples were analyzed for AMPA and GLY using the FMOC derivatization approach after MIP extraction; the rest were processed using the direct injection approach considering AMPA, GLY, and fosetyl as target compounds. Concentrations were estimated against matrix-matched standards for the FMOC derivatization protocol and using solvent-based calibration standards (ethanol:ultrapure water; 12:88), containing the surrogate standards at the same concentration as in the samples, for the direct method. Identification was based on retention time and transition ratios within maximum variations specified in SANTE/12682/2019 guide: ± 0.1 min for retention time and ± 30% regarding transition ratios. Moreover, a procedural blank was processed every five samples to detect possible contamination between samples.

**Fig. 4:**
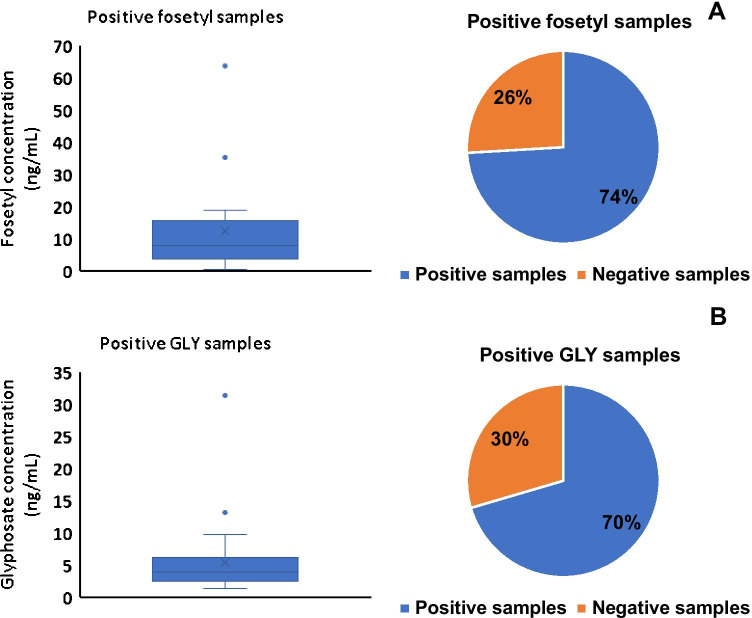
Box-whisker plots of fosetyl (**A**) and GLY (**B**) concentrations in wine samples

Fosetyl residues were found in a range of concentrations between 0.5 and 63.8 ng mL^−1^, counting 74% of positive samples (Fig. 4A) in a set of 27 wine samples processed using the polyvinyl alcohol column. Regarding GLY, the herbicide was found in 70% of 44 wines with concentrations ranging from 1.4 to 31.4 ng mL^−1^ (Fig. 4B). With the exception of two samples, GLY residues remained below 5 ng mL^−1^. Concentrations measured in each sample (average of duplicate measurements) are provided as supplementary information (Tables S2, S3). Finally, AMPA could be detected, at a concentration below LOQ, in just a wine sample, which contained the highest measured level of GLY (31.4 ng mL^−1^). Residues of GLY are of low significance compared to the MRL established by the EU for this herbicide in vinification grapes (500 µg kg^−1^) [23], and they are in agreement with those reported by other researchers. For example, Zoller et al. [17] reported a maximum residue of 13.2 ng mL^−1^ for GLY in a red wine in 2018. On the other hand, Rubio et al. (2016) confirmed the early detection of GLY residues in wine in the range from 2.6 to 29 ng mL^−1^ using immunoassay techniques [24]. Regarding fosetyl, concentrations shown in Fig. 4A are very low compared with the MRL of 100 mg kg^−1^ [25] set for vinification grapes; however, that value is defined for the sum of fosetyl (ethyl phosphonic acid) and phosphonic acid, whose quantification has not been included in the current study.

**Fig. 5:**
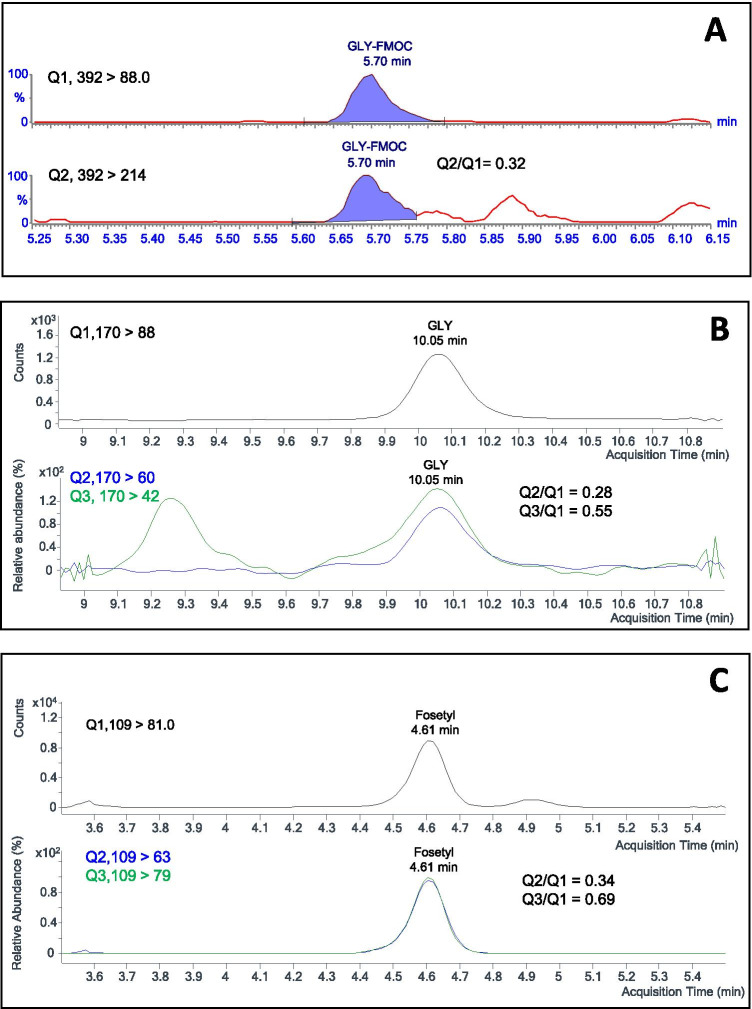
LC–ESI–MS/MS (MRM) chromatograms for quantification (Q1) and qualification (Q2 to Q3) transitions of GLY and fosetyl in non-spiked wine samples. **A** MRM traces (Q1, 392 > 88.0; Q2, 292 > 214.0) for GLY-FMOC in sample R12 (measured concentration 6.3 ng mL^−1^). **B** MRM traces for GLY (Q1, 170 > 88; Q2, 170 > 60, Q3, 170 > 42) in sample R23 (measured concentration 4.3 ng mL^−1^). **C** MRM (Q1, 109 > 81; Q2, 109 > 63; Q3, 109 > 79) traces for fosetyl in sample R23 (measured concentration 8.1 ng mL^−1^)

Figure 5 shows chromatograms for GLY and fosetyl in two non-spiked wine samples. Figure 5A displays the plots for Q1 and Q2 transitions of GLY-FMOC in a wine containing 6.3 ng mL^−1^ of the herbicide (sample code R12, Table S2). Figure 5B and C show the MRM chromatograms (including quantification, Q1, and qualification transitions, Q2 and Q3) for GLY and fosetyl, as non-derivatized compounds, in another wine (sample code R23) containing 4.1 and 8.1 ng mL^−1^ of the herbicide and fungicide transformation product, respectively.

## Conclusions

Application of the FMOC derivatization method to GLY and AMPA determination in wine samples requires the previous isolation of target compounds from the wine matrix to enhance the efficiency of their further derivatization. This process can be successfully performed employing a commercially available MIP sorbent, at the expense of introducing an extra step in the already tedious FMOC derivatization approach. The strong anionic exchange column tested in this research permitted the fast, effective separation of AMPA, GLY, and fosetyl without derivatization of the two former compounds. In combination with a top-range QqQ MS instrument, the three compounds can be determined in diluted wine matrices achieving procedural LOQs in the range from 0.4 to 8.3 ng mL^−1^. Even when the LOQ obtained for AMPA is higher than that achieved following the FMOC derivatization, the above values are deemed as suitable to evaluate the presence of the compound in wine samples at levels which guarantee the safety of the beverage. Analysis of wine samples reflected the often presence of GLY residues; however, 95% of samples above method LOQ contained residues of GLY below 5 ng mL^−1^. Such levels are of little relevance from the point of view of GLY exposure through regular wine consumption; however, they confirm the input of minimum amounts of the herbicide in the vinification process.

## Supplementary Information

Below is the link to the electronic supplementary material.

Supplementary file1 (DOCX 441 KB)
