# Supplementary material for: Approaches to liquid chromatography tandem mass spectrometry assessment of glyphosate residues in wine
Source: Anal Bioanal Chem. 2021 Nov 25;414(3):1445–55. doi: 10.1007/s00216-021-03775-w (PMC8724176; doi:10.1007/s00216-021-03775-w)
Supplement: Supplementary file 1 — Supplementary file1 (DOCX 441 KB) [file 216_2021_3775_MOESM1_ESM.docx]

Supplementary information to manuscript:

**Approaches to liquid chromatography tandem mass spectrometry assessment of glyphosate residues in wine**

L. Pérez-Mayán, G. Castro, M. Ramil, R. Cela, I. Rodríguez*

Department of Analytical Chemistry, Nutrition and Food Sciences. Research Institute on Chemical and Biological Analysis (IAQBUS). Universidade de Santiago de Compostela, 15782-Santiago de Compostela, Spain.

Table S1. Summary of chromatographic conditions employed during compounds determination by LC-MS.

| Determination approach | LC column | Separation mechanism | Supplier | Mobile phase A | Mobile phase B | Mobile phase flowrate  (mL min^-1^) | Column temperature  (º C) | Gradient |
| --- | --- | --- | --- | --- | --- | --- | --- | --- |
| FMOC-derivatization | Acquity BEH C18 | Reversed-phase | Waters | H_2_O, 0.5% FA | MeOH, 0.5% FA | 0.4 | 40 | 5% B (0 min);  60 % B (5.5 min); 100% B (7-9 min); 5% B (9.1-14 min) |
| Free compounds | Torus DEA | HILIC plus weak anion exchange | Waters | H_2_O, 20 mM NH_4_HCO_2_ | ACN, 0.9% FA | 0.5 | 30 | 90% B (0-0.2 min); 40% B (3-6.5 min); 90 % B (7-20 min) |
|  | Metrosep A Supp | Strong anionic exchange | Metrohm | H_2_O, 0.02% FA | ACN: H_2_O (1:1), 90 mM NH_4_CO_3_H | 0.35 | 30 | ^a^10% B (0-1.5 min); 20%B (1.6-3 min); 50%B (8-11 min); 100% B (11.5-16.5 min); 10%B (17-20 min) |
|  |  |  |  | H_2_O: ACN (1:1), 45 mM NH_4_CO_3_H | H_2_O, 50 mM NH_4_CO_3_H | 0.3 | 30 | ^b^0% B (0-2 min); 35% B (7 min); 60 % B (9.5 min); 100% B (11-16 min); 0% B (16.20- 23 min) |

^a^ Termed as gradient 1 in the manuscript

^b^ Termed as gradient 2 in the manuscript

Table S2. Concentration of GLY (ng mL^-1^) measured in commercial wine samples.

| **Sample code** | **Wine type** | **Concentration (ng mL^-1^)** |
| --- | --- | --- |
| W1 | White | 2.3 |
| W2 | White | 5.5 |
| W3 | White | 5.4 |
| W4 | White | 13.2 |
| W5 | White | 4.4 |
| W6 | White | 4.0 |
| W7 | White | 2.5 |
| W8 | White | 3.0 |
| W9 | White | n.d. |
| W10 | White | n.d. |
| R1 | Red | 3.4 |
| R2 | Red | 2.4 |
| R3 | Red | 3.4 |
| R4 | Red | 2.1 |
| R5 | Red | 2.8 |
| R6 | Red | 4.7 |
| R7 | Red | 1.4 |
| R8 | Red | 31.4 |
| R9 | Red | 5.9 |
| R10 | Red | 6.5 |
| R11 | Red | 9.8 |
| R12 | Red | 6.3 |
| R13 | Red | 6.3 |
| R14 | Red | 2.4 |
| R15 | Red | 4.2 |
| R16 | Red | 1.6 |
| R17 | Red | 3.1 |
| R18 | Red | 9.7 |
| R19 | Red | 2.3 |
| R20 | Red | 4.0 |
| R21 | Red | 3.7 |
| R22 | Red | 6.9 |
| R23 | Red | 4.1 |
| R24 | Red | n.d. |
| R25 | Red | n.d. |
| R26 | Red | < LOQ |
| R27 | Red | n.d. |
| R28 | Red | < LOQ |
| R29 | Red | < LOQ |
| R30 | Red | < LOQ |
| R31 | Red | n.d. |
| R32 | Red | n.d. |
| R33 | Red | n.d. |
| R34 | Red | n.d. |

Table S3. Concentration of Fosetyl (ng mL^-1^) found in wine samples.

| **Sample code** | **Wine type** | **Concentration (ng mL^-1^)** |
| --- | --- | --- |
| W3 | White | 0.5 |
| W7 | White | < LOQ |
| W10 | White | 63.8 |
| R1 | Red | 11.2 |
| R2 | Red | 15.3 |
| R3 | Red | 2.8 |
| R4 | Red | n.d. |
| R5 | Red | 7.9 |
| R6 | Red | 16.1 |
| R7 | Red | 3.6 |
| R8 | Red | n.d. |
| R10 | Red | 3.7 |
| R14 | Red | 15.9 |
| R15 | Red | 35.5 |
| R16 | Red | 4.9 |
| R17 | Red | n.d. |
| R18 | Red | 13.1 |
| R19 | Red | 8.3 |
| R23 | Red | 8.0 |
| R24 | Red | n.d. |
| R25 | Red | 19.0 |
| R26 | Red | 4.8 |
| R28 | Red | n.d. |
| R29 | Red | n.d. |
| R30 | Red | 5.9 |
| R32 | Red | 12.0 |
| R34 | Red | 1.2 |

Fig. S1. Pictures of a red wine sample following different pre-treatments before FMOC derivatization. A, 1:5 dilution with ultrapure water. B, 1:5 diluted wine adjusted at pH 9 with borate buffer; c) MIP aqueous extract from red wine (dilution factor 1:5); d) MIP aqueous extract neutralized and adjusted to pH 9.

Fig. S2. Normalized responses for time-course of the FMOC derivatization process using MIP extracts form spiked red wine.

Fig. S3. Normalized responses obtained for the FMOC derivatives of AMPA and GLY in spiked (20 ng mL^-1^) aliquots of a white wine sample using in-sample derivatization versus those obtained considering MIP extraction-purification before FMOC derivatization, n=3 replicates.

Fig. S4. Structures assigned to product ions in the MS/MS spectra of GLY (A) and AMPA (B) as FMOC derivatives.
